# Evaluating Tumor Burden as a Predictive Biomarker for Epidermal Growth Factor Receptor Targeted Kinase Inhibitor Therapy in Advanced Non–Small Cell Lung Cancer

**DOI:** 10.1200/PO-25-00884

**Published:** 2026-04-09

**Authors:** Rika Terashima, Judy Fan, Fatma Gunturkun, Grant Nieda, Xiaomei Fan, Emily M. Rodriguez, Annabel X. Tan, Stefan Thottunkal, Maggie Shaw, Chloe C. Su, Aparajita Khan, Victoria Y. Ding, Ingrid Luo, Mina Satoyoshi, Archana Bhat, Bo Gu, Solomon M. Henry, Timothy J. Ellis-Caleo, Michelle Odden, Allison W. Kurian, Joel W. Neal, Heather A. Wakelee, Julie T. Wu, Summer S. Han

**Affiliations:** ^1^ Department of Medicine, Jacobi Medical Center, Albert Einstein College of Medicine, Bronx, NY; ^2^ Department of Epidemiology and Population Health, Stanford University School of Medicine, Stanford, CA; ^3^ Quantitative Sciences Unit, Stanford University School of Medicine, Stanford, CA; ^4^ Department of Computer Science and Engineering, Indian Institute of Technology (BHU) Varanasi, Varanasi, India; ^5^ Technology and Digital Solutions (TDS), Research Technology, and Research Data Services, Stanford Health Care and Stanford University School of Medicine, Stanford, CA; ^6^ Division of Oncology, Department of Medicine, Stanford University School of Medicine, Stanford, CA; ^7^ Stanford Cancer Institute, Stanford University, Stanford, CA; ^8^ Department of Neurosurgery, Stanford University School of Medicine, Stanford, CA

## Abstract

**PURPOSE:**

As treatment options for advanced non–small cell lung cancer (NSCLC) evolve, biomarkers are needed to guide therapy selection while balancing efficacy and toxicity. Although tumor burden is a promising candidate, its prognostic role in guiding epidermal growth factor receptor (EGFR)-targeted kinase inhibitor (TKI) therapies remains understudied in real-world settings.

**METHODS:**

We identified patients with de novo stage IV *EGFR*-mutant NSCLC treated with first-line EGFR-TKI at Stanford Health Care (2000-2021). Tumor burden metrics were manually annotated from 592 baseline radiology reports, encompassing size, number, and location (1,807 lesions). Multivariable Cox regression evaluated associations between tumor burden metric and overall survival (OS), adjusting for confounders, in the overall cohort and an osimertinib subgroup. A weighted composite tumor burden score was constructed using statistically significant metrics to stratify risk.

**RESULTS:**

Of 312 patients, bone metastasis (hazard ratio (HR)_adjusted_, 1.64 [95% CI, 1.23 to 2.19]) and the number of metastatic organs (HR_adjusted_, 1.21 [95% CI, 1.10 to 1.32]) were independently associated with worse OS and used to construct the composite score. Patients with low tumor burden (composite-score ≤ median 1.06) experienced better OS than those with high tumor burden, with a 3-year OS of 59.8% versus 41.5% (*P* = .001). Consistent findings were observed in the osimertinib subgroup, with a 3-year OS of 62.2% versus 44.6% (*P* = .03) for low versus high tumor burden.

**CONCLUSION:**

Tumor burden may serve as a prognostic biomarker in advanced NSCLC receiving EGFR-TKIs. These findings raise the hypothesis that durable survival in low-burden patients may be achievable with monotherapy, potentially sparing unnecessary toxicity from combination regimens. This warrants prospective validation comparing monotherapy versus combination strategies.

## INTRODUCTION

Lung cancer remains the leading cause of cancer-related mortality worldwide,^1^ with non–small cell lung cancer (NSCLC) comprising approximately 85% of all cases.^2^ In recent years, the treatment landscape for advanced NSCLC has evolved dramatically with the introduction of immunotherapies targeting PD-1/PD-L1 and targeted therapies against oncogenic drivers such as *EGFR*, *ALK*, and *ROS1*.^3-8^

**CONTEXT**

**Key Objective**
Can baseline tumor burden from routine radiology reports be summarized into a composite score that prognosticates overall survival (OS) in de novo stage IV *EGFR*-mutant non–small cell lung cancer treated with first-line epidermal growth factor receptor (EGFR) targeted kinase inhibitors (TKIs), beyond single-metric or trial-/RECIST-limited assessments?
**Knowledge Generated**
In 312 patients, bone metastasis (hazard ratio [HR], 1.64) and the number of metastatic organs (HR, 1.21 per organ) were independently associated with OS and were combined into a weighted composite score. Low versus high composite burden stratified 3-year OS in the full cohort (59.8% *v* 41.5%) and the osimertinib subgroup (62.2% *v* 44.6%).
**Relevance**
The composite score uses information commonly available in baseline imaging reports and could be applied to risk stratify patients starting EGFR TKI therapy, supporting clinical management discussions and informing future studies of treatment selection strategies.

As treatment strategies become increasingly complex,^3^ the need for reliable biomarkers to guide therapeutic decisions has grown more urgent.^9,10^ Biomarkers play a critical role in identifying patients most likely to benefit from specific therapies while helping to minimize unnecessary toxicity and cost.^9^ In advanced NSCLC, PD-L1 guides first-line immunotherapy with or without chemotherapy.^11^ Patients with high PD-L1 may avoid chemotherapy, whereas low PD-L1 often benefits from combination therapy. Yet, responses vary widely, underscoring the need for additional predictive biomarkers. Tumor burden has emerged as one of the clinically relevant biomarkers^12-16^ as there is wide variation in disease extent which can influence the chance of response. For example, in the context of immunotherapy, patients with a high tumor burden have been suggested to receive first-line immunotherapy with chemotherapy combination therapy, whereas those with low burden have been suggested to receive immunotherapy monotherapy,^17^ who can reduce their exposure to toxicity.

Despite its growing significance, systematic evaluation of tumor burden as a biomarker for epidermal growth factor receptor (EGFR) targeted kinase inhibitors (TKIs) remains limited, even as first-line options expand.^18,19^ EGFR TKIs are central for EGFR-mutant NSCLC, which is common in never smokers and individuals of East Asian descent.^20^ Earlier-generation TKIs (eg, gefitinib, erlotinib) improved outcomes, but resistance led to third-generation agents such as osimertinib; after the 2018 FLAURA trial, osimertinib became standard first-line therapy.^21^ More recently, combination strategies have emerged as potential approaches to further improve outcomes, though at the cost of added toxicity. In FLAURA2, osimertinib-chemotherapy improved progression-free survival (PFS) versus osimertinib alone but increased grade ≥3 adverse events (64% *v* 27%) and treatment-related deaths (5 *v* 1).^22^ MARIPOSA similarly improved outcomes with amivantamab-lazertinib but raised grade ≥3 toxicity (75% *v* 43%).^23^ These data highlight the need to identify patients who may safely receive monotherapy, such as those with low tumor burden, although defining these patients remains unclear.

While several studies^24-26^ have examined tumor burden in EGFR TKI–treated NSCLC, many are limited by treatment era, setting, or how burden is measured, reducing real-world generalizability. Cha et al^24^ evaluated tumor burden in 2015 but did not include osimertinib and largely analyzed later-line TKI use, where prior therapies may confound tumor burden as an independent prognostic factor. More recent post hoc analyses from FLAURA2^25^ and other studies^26^ have incorporated tumor burden, yet assessments are often constrained by trial data collection and emphasize RECIST-based measures, with limited capture of unmeasured lesions. In addition, trial populations may not fully represent routine clinical practice.

This study aims to leverage real-world data to comprehensively evaluate tumor burden as a potential biomarker for response to first-line EGFR TKI therapy, specifically osimertinib, in stage IV de novo *EGFR*-mutant NSCLC. We analyze various parameters such as lesion size, number, and location to establish a composite score that stratifies patients into low- and high-tumor burden groups. By identifying a subgroup of patients with low tumor burden who may achieve favorable outcomes with TKI monotherapy, we aim to identify patients who may safely forgo the added toxicity of combination therapy.

## METHODS

### Study Cohort and Data Sources

**FIG 1.**
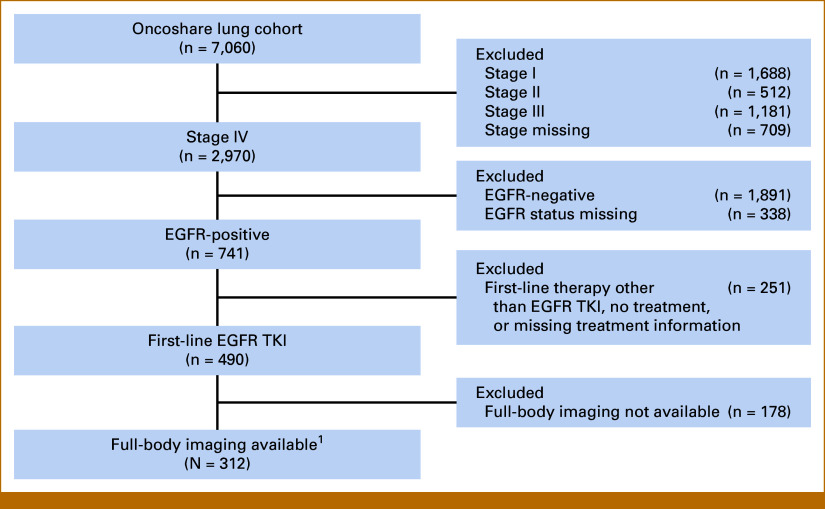
Flowchart of cohort selection. EGFR, epidermal growth factor receptor; TKI, targeted kinase inhibitor.

We used data from Oncoshare-Lung, an electronic health record (EHR)–based cohort^27^ from Stanford Health Care. This resource contains clinical and imaging data for oncology research in Northern California, enriched with data from Asian nonsmoker patients with lung cancer. The study included 312 patients with pathologically confirmed de novo stage IV NSCLC who received first-line EGFR TKI therapy, with no prior systemic therapy. Of 7,060 patients with lung cancer in Oncoshare-Lung, we excluded patients with stage I to III disease (n = 3,381) or missing stage information (n = 709), those who never tested positive for EGFR mutation (n = 2,229), those who received first-line therapy other than an EGFR TKI or had missing treatment data (n = 251), and those without imaging that included both chest and abdomen (n = 178). This resulted in a final analytic cohort of 312 patients (Fig 1). We restricted the cohort to de novo stage IV disease to ensure a homogeneous population initiating systemic, thereby minimizing confounding from prior early-stage management.^28^

### Clinical Note Selection and Tumor Burden Assessment

A total of 592 notes (280 brain MRI or head CT reports and 312 positron emission tomography/computed tomography [PET/CT] or full-body CT reports) were selected for manual annotations across 312 patients. For each patient, we selected one full-body imaging study (PET/CT or CT of the chest, abdomen, and pelvis) and one brain imaging study, preferably brain MRI (Data Supplement, Method S1).

Three trained annotators (R.T., J.F., X.F.) manually reviewed the reports to extract tumor burden metrics using a standardized annotation process (see the Data Supplement, Method S1, for guidelines). They extracted lesion size, count, anatomic location, and radiologic descriptors indicative of malignancy from one full-body and one brain imaging report per patient. The curated tumor metrics included sum of tumor sizes, maximum tumor size, the number of lesions, the number of metastatic organs, and specific metrics for each metastatic organ. Metastatic organ categories included lungs, lymph nodes, liver, adrenal glands, kidneys, spleen, pancreas, bone, brain, and others. Only lesions attributable to NSCLC were included, following detailed protocols to ensure consistency. This process resulted in 11 baseline tumor burden variables, listed in Table 2.

### Study Outcomes

The primary outcome was overall survival (OS), defined as the time from diagnosis to death from any cause or censoring on October 31, 2023. Sensitivity analysis was conducted using the start date of treatment as time zero for survival analysis.

### Treatment and Clinical Variables

To evaluate patient characteristics, we extracted the following clinical data: Age at diagnosis, sex, race, smoking status, stage, histology, primary tumor location, Eastern Cooperative Oncology Group performance status (ECOG PS), EGFR agent, combination therapy, treatment delay, and time to death. All demographic variables besides ECOG PS were extracted from Oncoshare-Lung which curated the data from structured EHRs.^27^ ECOG PS was manually abstracted from oncologist progress notes because of its inconsistent availability in structured fields. First-line and second-line treatment data were obtained from the Oncoshare-Lung database, EHR pharmacy table, and clinical notes. For all patients included in our analysis, treatment information was verified through manual review of oncologist progress notes to ensure accuracy.

### Statistical Analysis

To evaluate the association between OS and each tumor burden metric, we conducted multivariable Cox regression analyses as the primary analysis, adjusting for covariates including age at diagnosis, ECOG PS, sex, and delay to treatment. These covariates were selected a priori based on the literature^29-33^ and multivariate Cox regression for OS (Data Supplement, Table S3). To account for multiple tests on 18 tumor burden metrics (11 baseline and seven transformed metrics, Data Supplement, Method S2), we applied the Bonferroni method with α = .00278 (0.05/18) for statistical significance. For missing data, we assessed the missing rate for each baseline patient covariate (Data Supplement, Table S1). With a small missing rate (<2.2%), we performed a complete case analysis for the Cox regression. Kaplan-Meier curves were generated to evaluate survival patterns in the overall population and by key variables identified in the multivariable analyses.

### Constructing a Composite Tumor Burden Score

Based on statistically significant tumor metrics (*P* < .00278 from the Bonferroni correction) identified through a set of multivariable Cox regression models, we constructed a composite tumor burden score as the weighted sum of these variables. We used the regression coefficients of each tumor burden metric in the adjusted Cox regression as weights, similar to constructing a polygenic risk score in genetic studies.^34^ For example, if two variables, X1 and X2, remain significant after adjustment, with coefficients beta1 and beta2, the tumor burden score can be expressed as tumor burden score = beta1 * (X1) + beta2 * (X2). We used the median value of the composite tumor burden score to determine the cutoff for high versus low tumor burden (see the Data Supplement, Method S3, for details).

### Sensitivity Analysis and Subgroup Analysis

We conducted sensitivity and subgroup analyses to evaluate the robustness of our main findings. First, we focused on patients who received osimertinib as the first-line EGFR TKI, given its clinical relevance. We developed a composite tumor burden variable using weights from multivariable Cox models in this subgroup, with the median composite score serving as the cutoff for high versus low tumor burden. Second, we performed a sensitivity analysis using treatment start date as time zero for Cox regression analysis of OS. Third, recognizing that *EGFR* mutation type affects survival, we stratified by mutation type, comparing patients with exon 19 deletions with those with exon 21 L858R mutations^35,36^ (p19). Within each subgroup, we conducted multivariable Cox regression, adjusting for age at diagnosis, ECOG PS, sex, and delay to treatment, using diagnosis date as time zero. Fourth, we performed a sensitivity analysis further adjusting for whether patients received second-line therapy (status and its timing as a time-varying covariate). Finally, we performed univariate Cox regression analyses. More details are given in the Data Supplement (Method S4).

## RESULTS

Baseline characteristics of the study cohort are summarized in Table 1. The patients in this cohort were predominantly female (62.2%), Asian (57.7%), and never smokers (70.2%), with a mean age of 65 years and 95% adenocarcinoma histology. A total of 123 patients (39.4%) were treated with osimertinib as first-line therapy, 183 (58.7%) with erlotinib, four (1.3%) with afatinib, and two (0.6%) with gefitinib. No patients received EGFR TKI combined with traditional chemotherapy. Among patients with available follow-up after first-line EGFR TKI therapy, 66.3% received subsequent systemic therapy, with a median time to next treatment of 375 days (IQR, 233.0-718.0).

**TABLE 1.** Baseline Characteristics of the Patients Who Received First-Line EGFR TKI in the Overall Cohort and the Subgroup of Patients Who Received Osimertinib as First-Line EGFR TKI

Characteristic	Total (N = 312)	Osimertinib (n = 123)
Age, years		
Mean (SD)	65.0 (12.5)	66.3 (11.5)
Median, IQR (Q1-Q3)	65.9 (56.2-73.8)	67.2 (58.3-74.5)
Sex, No. (%)		
Female	194 (62.2)	76 (61.8)
Male	118 (37.8)	47 (38.2)
Asian, No. (%)		
No	128 (41.0)	46 (37.4)
Yes	180 (57.7)	75 (61.0)
Missing	4 (1.3)	2 (1.6)
Smoking status, No. (%)		
Never smoker	219 (70.2)	87 (70.7)
Ever smoker	72 (23.1)	28 (22.8)
Missing	21 (6.7)	8 (6.5)
Stage, No. (%)		
IV	312 (100)	123 (100)
Adenocarcinoma, No. (%)		
No	19 (6.1)	4 (3.3)
Yes	293 (93.9)	119 (96.7)
Primary tumor location, No. (%)		
Lower	79 (25.3)	35 (28.5)
Upper	164 (52.6)	62 (50.4)
Other	69 (22.1)	26 (21.1)
ECOG performance status, No. (%)		
0-1	248 (79.5)	99 (80.5)
2+	57 (18.3)	19 (15.4)
Missing	7 (2.2)	5 (4.1)
*EGFR* mutation type,^b^ No. (%)		
Exon 19 deletion	157 (48.5)	54 (41.5)
Exon 21 L858R	131 (40.4)	60 (46.2)
Other	25 (7.7)	14 (10.8)
Missing	11 (3.4)	2 (1.5)
Compound EGFR mutations, No. (%)		
No	289 (92.6)	114 (92.7)
Yes	12 (3.8)	7 (5.7)
Missing	11 (3.5)	2 (1.6)
First-line therapy, No. (%)		
EGFR agent		
Osimertinib	123 (39.4)	123 (100)
Erlotinib	183 (58.7)	0 (0)
Afatinib	4 (1.3)	0 (0)
Gefitinib	2 (0.6)	0 (0)
Combination therapy		
No	306 (98.1)	123 (100)
Yes^a^	6 (1.9)	0 (0)
Treatment delay (days)		
Mean (SD)	37.9 (29.5)	40.1 (33.6)
Median, IQR (Q1-Q3)	31.5 (21.8-44.2)	31.0 (22.0-45.0)
Second-line therapy		
Received		
No	105 (33.7)	60 (48.8)
Yes	207 (66.3)	63 (51.2)
Treatment duration (days)^c^		
Mean (SD)	331.5 (363.0)	258.2 (283.6)
Median, IQR (Q1-Q3)	218 (94.5-439.0)	143.5 (71.0-302.5)
Time to treatment (days)^d^		
Mean (SD)	561.7 (471.4)	574.0 (444.3)
Median, IQR (Q1-Q3)	408.0 (255.0-760.0)	400.0 (300.0-826.0)
Time to death (days)		
Mean (SD)	930.1 (652.3)	769.2 (553.7)
Median, IQR (Q1-Q3)	805.0 (425.0-1,264.0)	627.5 (286.0-1,132.8)

Abbreviations: ECOG, Eastern Cooperative Oncology Group; EGFR, epidermal growth factor receptor; SD, standard deviation; TKI, targeted kinase inhibitor.

^a^ All six patients received first-line EGFR TKI in combination with momelotinib.

^b^ Patients with compound EGFR mutations were counted in each applicable mutation category.

^c^ Treatment duration was calculated among patients who initiated and completed second-line therapy (n = 183 for the entire cohort, n = 54 for the osimertinib subgroup).

^d^ Time to treatment was calculated from the diagnosis date to the second-line therapy start date.

**TABLE 2.** Summary Statistics of Various Tumor Burden Metrics in the Overall Cohort and in the Subgroup of Patients Who Received Osimertinib as First-Line EGFR TKI

Characteristic	Total (N = 312)	Osimertinib (n = 123)
Sum of tumor sizes (cm)		
Mean (SD)	8.9 (5.6)	9.1 (5.7)
Median IQR (Q1-Q3)	7.7 (4.8-11.6)	8.2 (5.3-11.5)
Maximum tumor sizes (cm)		
Mean (SD)	3.7 (2.0)	3.8 (2.1)
Median, IQR (Q1-Q3)	3.4 (2.4-4.7)	3.4 (2.3-5.0)
No. of lesions		
Mean (SD)	5.8 (2.9)	5.7 (2.7)
Median, IQR (Q1-Q3)	6.0 (4.0-8.0)	5.0 (4.0-7.0)
No. of metastatic organs		
Mean (SD)	3.7 (1.5)	3.9 (1.4)
Median, IQR (Q1-Q3)	4.0 (3.0-5.0)	4.0 (3.0-5.0)
>3 metastatic organs, No. (%)		
Yes	166 (53.2)	68 (55.3)
No	146 (46.8)	55 (47)
Brain metastasis, No. (%)		
Yes	154 (49.4)	68 (55.3)
No	150 (48.1)	52 (42.3)
Resected	2 (0.6)	0 (0)
Missing	6 (1.9)	3 (2.4)
Sum of brain lesion sizes (cm)		
Mean (SD)	1.3 (2.4)	1.6 (2.7)
Median, IQR (Q1-Q3)	0.0 (0.0-1.8)	0.3 (0.0-2.2)
Maximum brain lesion sizes (cm)		
Mean (SD)	0.6 (1.0)	0.7 (1.0)
Median, IQR (Q1-Q3)	0.0 (0.0-0.9)	0.2 (0.0-1.0)
Liver metastasis, No. (%)		
Yes	60 (19.2)	25 (20.3)
No	252 (80.8)	98 (79.7)
Bone metastasis, No. (%)		
Yes	169 (54.2)	65 (52.8)
No	143 (45.8)	58 (47.2)
Adrenal metastasis, No. (%)		
Yes	264 (84.6)	99 (80.5)
No	48 (15.4)	24 (19.5)

Abbreviations: EGFR, epidermal growth factor receptor; SD, standard deviation; TKI, targeted kinase inhibitor.

**FIG 2.**
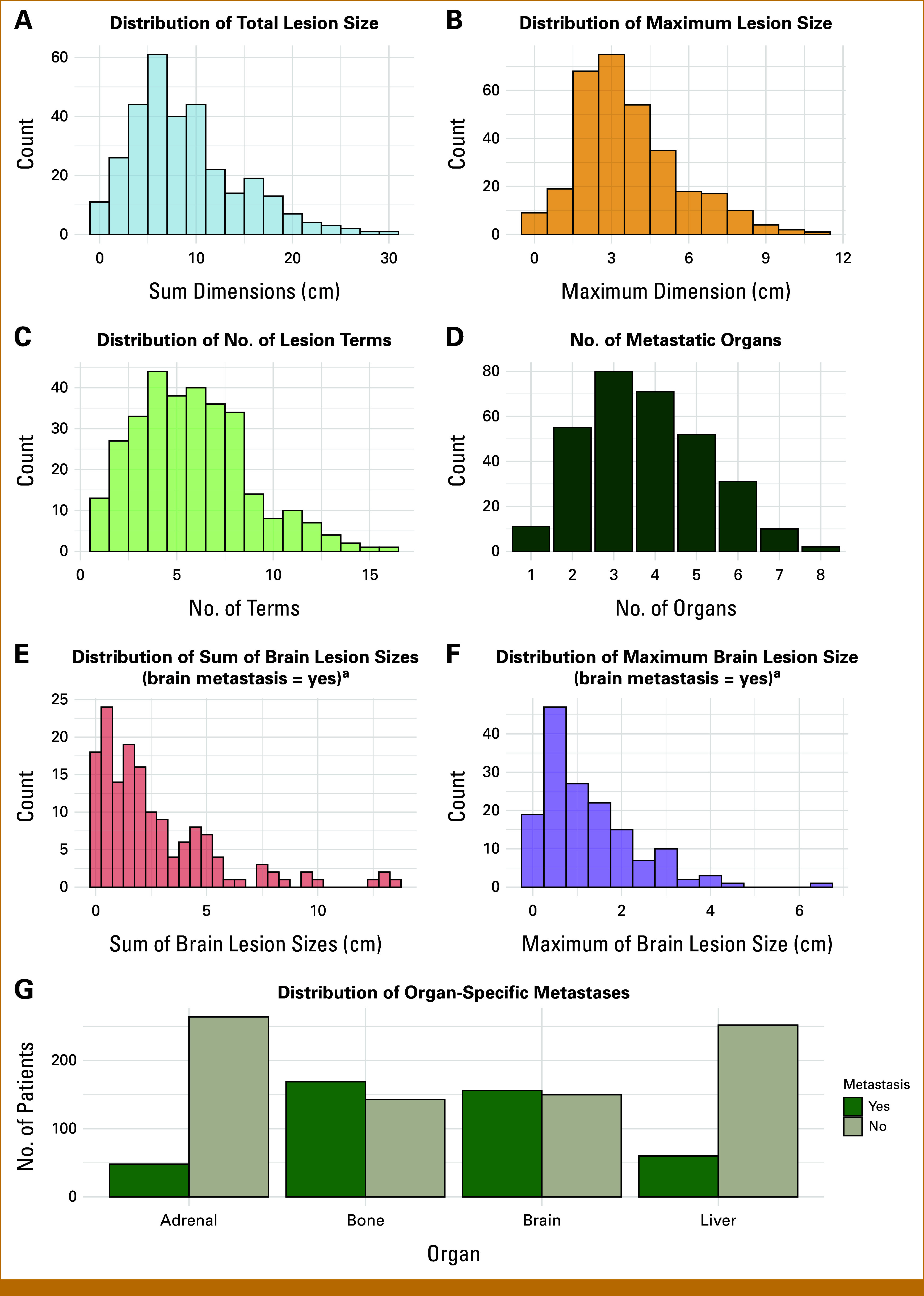
Distribution of tumor burden variables among 312 patients with NSCLC receiving first-line EGFR TKI. (A) Total metastatic lesion size (sum of all lesion dimensions) in centimeters per patient. (B) The maximum single metastatic lesion dimension (cm) per patient. (C) The number of metastatic lesions per patient. (D) Bar plot showing the distribution of the number of metastatic organ sites per patient. (E) Sum brain lesion size (cm) among patients with brain metastases. (F) Maximum brain lesion size (cm) among patients with brain metastases. (G) Bar plot of metastasis presence (yes/no) across key organ systems: adrenal glands, bone, brain, and liver. ^a^Excluding missing and counting surgically resected cases as “Yes.” EGFR, epidermal growth factor receptor; NSCLC, non–small cell lung cancer; TKI, targeted kinase inhibitor.

The summary statistics of the curated comprehensive tumor burden metrics—including sum of tumor sizes, maximum tumor size, the number of lesions, the number of metastatic organs, >3 metastatic organ, brain metastasis status, sum of brain lesion sizes, maximum brain lesion size, liver metastasis status, bone metastasis status, and adrenal metastasis status—are shown in Table 2 and visualized in Figure 2. Notably, 52.6% of patients had metastases in more than three organ systems. Brain metastases were present in 48.1% of the cohort, and liver and bone metastases were identified in 19.2% and 54.2% of patients, respectively. The median sum of tumor sizes was 7.7 cm (IQR, 4.8-11.3), with a median maximum tumor size of 3.4 cm (IQR, 2.4-4.7). Patients had a median of six lesions (IQR, 4.0-8.0) and a median of four metastatic organs (IQR, 3-5).

A series of multivariable Cox regression analyses revealed that two tumor burden metrics—the number of metastatic organs and the presence of bone metastasis—were statistically significantly associated with OS after receiving first-line EGFR TKI in this cohort (Data Supplement, Table S5 and Table S4 for univariate analysis). For each additional involved metastatic organ, patients experienced a 21% increase in the risk of overall mortality (hazard ratio [HR]_adjusted_, 1.21 [95% CI, 1.10 to 1.32]; *P* = .00007; Data Supplement, Table S5). Similarly, patients with bone metastasis had a 64% increase in the risk of overall mortality compared with those without bone involvement (HR_adjusted_, 1.64 [95% CI, 1.23 to 2.19]; *P* = .00068; Data Supplement, Table S5) after receiving first-line EGFR TKI.

**FIG 3.**
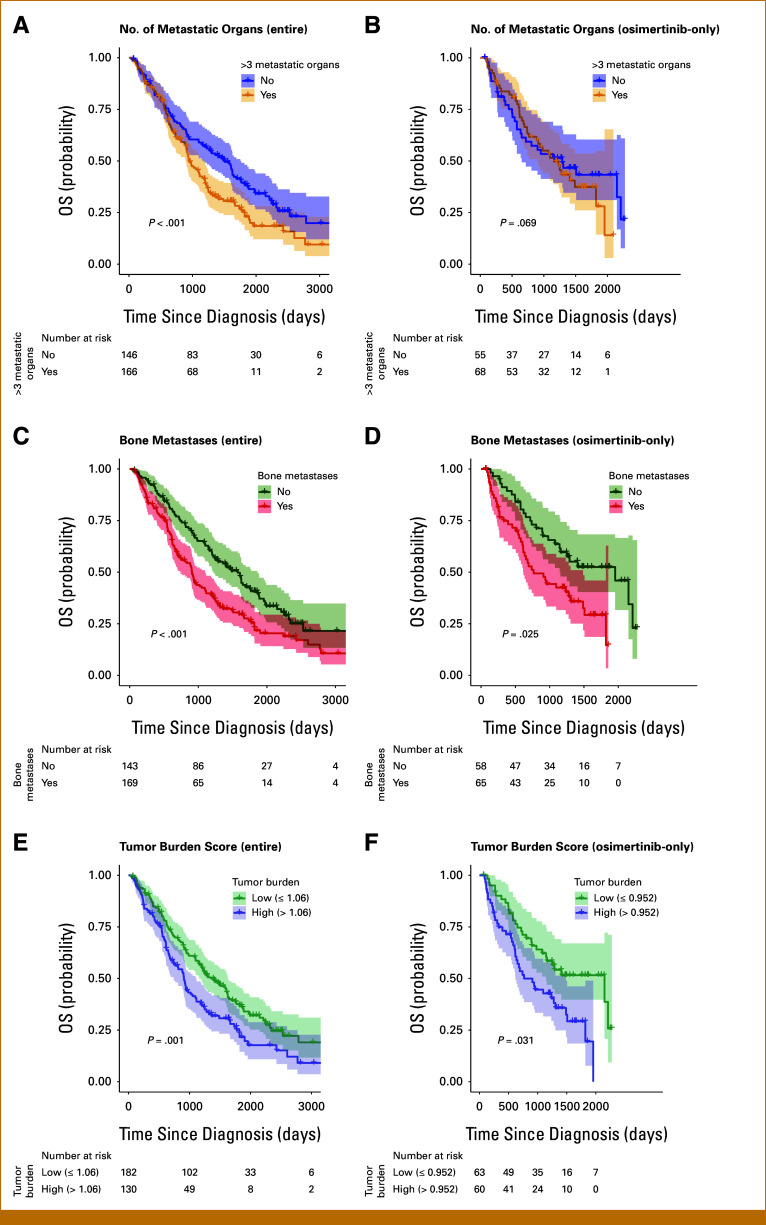
OS stratified by key tumor burden metrics in the entire cohort and among the subgroup of patients receiving osimertinib. Two statistically significant tumor burden metrics—the number of metastatic organs and the presence of bone metastasis—were identified after adjusting for multiple testing corrections in multivariable Cox regression. These metrics were used for the stratified analysis of OS, along with a composite tumor burden score based on the weighted sum of these two variables. The *P* value in each survival curve panel represents the *P* value from the adjusted multivariable Cox regression. The following stratifications were applied for each panel: (A) and (B) Number of metastatic organs (>3 *v* ≤3, treated as continuous in the Cox regression model), (C) and (D) presence of bone metastases, and (E) and (F) A composite tumor burden score dichotomized at the median (high >1.06 *v* low ≤1.06 for the total cohort; high >0.952 *v* low <0.952 for the osimertinib subgroup). OS, overall survival.

Using the two statistically significant metrics after adjusting for multiple testing (*P* < .00278), that is, the number of metastatic organs and the presence of bone metastasis, we constructed a weighted composite tumor burden score (Methods and Data Supplement, Method S3). When we stratified the study cohort by the median (>1.06) of the composite tumor burden score, we found that patients with low tumor burden (ie, tumor burden score ≤1.06, median) had a better OS after receiving first-line EGFR TKI compared with those with high tumor burden (ie, >1.06; Fig 3D, Data Supplement, Fig S1). Specifically, the 3-year OS for the low-tumor burden group was 59.8% (95% CI, 52.9 to 67.6) versus 41.5% (95% CI, 33.5 to 51.3) for the high-tumor burden group (*P* = .001 based on adjusted Cox regression) in the overall cohort. Patient characteristics stratified by tumor burden group are shown in the Data Supplement (Table S2).

**FIG 4.**
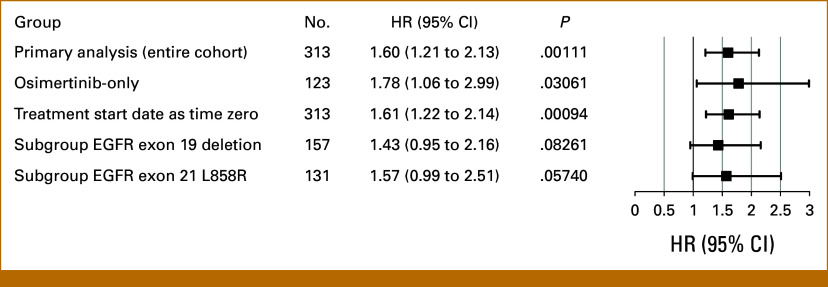
Forest plots for association between overall survival and composite tumor burden score in the entire cohort and by sensitivity analyses. Each multivariable Cox regression model (represented by each single line) was adjusted for age at diagnosis, ECOG performance status (2+ *v* 0-1), sex, and delay to treatment. ECOG, Eastern Cooperative Oncology Group; EGFR, epidermal growth factor receptor; HR, hazard ratio.

Subgroup analysis focusing on patients who received osimertinib for first-line EGFR TKI demonstrated results that were generally consistent with those observed in the entire cohort (Figs 3B, 3D and 3F, and 4). Notably, patients with low tumor burden (ie, tumor burden score ≤0.94, median estimated based on this subgroup) experienced better OS after receiving first-line osimertinib, with a 3-year OS of 62.2% (95% CI, 50.9 to 76.1) compared with 44.6% (95% CI, 33.4 to 59.6) for the high-tumor burden group (*P* = .03 based on adjusted Cox regression; Fig 3F).

Sensitivity analyses using the treatment initiation date (rather than diagnosis date) as the time zero for the outcome of OS, inclusion of second-line therapy (yes/no) as a time-varying covariate, and subgroup analyses stratified by *EGFR* mutation type all yielded consistent results (Data Supplement, Fig S2 and Fig 4). Across all analyses, patients with low composite tumor burden demonstrated superior OS compared with those with high tumor burden. The direction of the association between OS and composite tumor burden remained consistent in both the exon 19 deletion (n = 157) and exon 21 L858R (n = 131) subgroups although statistical significance was attenuated because of the smaller sample sizes in these subgroups.

## DISCUSSION

In this real-world study of stage IV de novo *EGFR*-mutant NSCLC, we evaluated tumor burden as a potential biomarker for predicting treatment response to EGFR TKI. We found that a weighted composite score—integrating metastatic organ count and bone metastasis—strongly predicted OS, particularly among patients receiving first-line osimertinib. Specifically, patients with low tumor burden (score ≤1.06) demonstrated significantly superior survival. This finding was consistently observed among patients who received osimertinib as first-line therapy. These results highlight the prognostic relevance of the composite tumor burden score in the osimertinib-treated population, generating the hypothesis that patients with low baseline tumor burden may achieve durable benefit with EGFR TKI monotherapy and could potentially avoid added toxicity from intensified regimens. Future prospective studies, including those comparing monotherapy and combination approaches (eg, FLAURA2-type strategies), are needed.

To the best of our knowledge, this study presents the first real-world investigation to comprehensively evaluate tumor burden—including lesion size, number, and anatomic location—as a potential biomarker for predicting OS in response to EGFR TKI, including osimertinib. We developed a standardized annotation process to measure various tumor burden parameters, establishing specific rules for determining lesion size and anatomic location through extensive iteration and validation by clinically trained evaluators. These standardized methods provide a reproducible framework for assessing tumor burden in clinical practice, addressing the lack of standardization in its measurement and definition. In contrast to studies that focus on single metrics and/or RECIST-limited trial measures, our composite tumor burden score, which incorporates the number of metastatic organs and the presence of bone metastasis, offers a practical and reproducible approach for stratifying patients based on baseline disease burden. Finally, our analysis adjusted for several key confounding factors, revealing that both ECOG performance status and delay to treatment were associated with OS in this cohort, underscoring the importance of accounting for clinical covariates when evaluating the prognostic value of tumor burden metrics.

While prior studies have linked greater tumor burden to worse outcomes with EGFR TKIs,^24-26^ the evidence base remains heterogeneous in how tumor burden is defined and in how directly findings translate to routine practice. Much of the earlier literature was generated in preosimertinib^24^ or later-line treatment settings.^25^ Other reports have focused on single-site or single-metric associations,^12,26,37^ and even contemporary trial-based analyses often emphasize RECIST target lesions,^25^ potentially under-representing clinically important disease such as RECIST-unmeasured or traditionally nonmeasurable metastases. Crucially, our real-world data capture these missing metrics; for example, we identify bone lesions—traditionally considered RECIST nonmeasurable—as a significant negative prognostic factor, a finding supported by other population-based studies^38^ and meta-analyses.^39,40^ By integrating these comprehensive metrics into a composite score within a modern first-line osimertinib cohort, our study specifically addresses the prognostic gaps left by trial-restricted data sets and outdated treatment eras.

Beyond the anatomic extent, tumor burden is a multidimensional construct encompassing mutation load, metabolic activity, and temporal dynamics.^15^ Emerging data link burden to circulating tumor DNA for monitoring resistance^41^ and fluorodeoxyglucose-PET for assessing metabolic volume, often outperforming static RECIST.^42^ Furthermore, anatomic location critically influences drug penetration.^43^ However, high costs and the need for prospective validation hinder the routine implementation of these advanced metrics. By contrast, our study presents an accessible composite score derived from standard clinical data. This approach offers a practical, complementary tool for decision making that aligns with emerging evidence without requiring additional, resource-intensive testing.

Despite its strengths, our study has several limitations. First, tumor size reporting in clinical radiology reports lacked standardization; while some reports included detailed measurements for all lesions, others documented only the largest findings. Second, our analysis focused exclusively on de novo stage IV NSCLC to avoid confounding by prior therapies, given that survival differs significantly in patients with distant recurrence.^28^ However, investigating tumor burden in recurrent populations remains critical to fully understand its prognostic value. Third, PFS could not be reliably abstracted retrospectively because of inconsistent documentation. Consequently, we descriptively reported subsequent systemic therapy and time to subsequent treatment, acknowledging that these metrics may differ from PFS because of factors like toxicity-driven switches or local treatments without systemic change. Finally, the proposed tumor burden score requires external validation in independent cohorts to ensure generalizability. Future research is essential to firmly establish tumor burden as a biomarker for clinical decision making. Specifically, prospective trials should stratify patients by baseline tumor burden and randomly assign them to EGFR TKI monotherapy or combination therapy to test the hypothesis that high tumor burden identifies those benefiting most from intensification.

In conclusion, our study shows that tumor burden, especially the number of metastatic organs and bone metastasis, is a strong predictor of OS in patients with de novo stage IV EGFR-mutant NSCLC receiving first-line EGFR TKI therapy. The composite tumor burden score is practical and clinically relevant, emphasizing the importance of baseline disease burden in prognostication. Future studies are needed to validate this score and determine its role in guiding treatment decisions for intensified strategies in EGFR-mutant NSCLC.
